# Mycosis fungoides with pseudocarcinomatous hyperplasia masquerading as verrucous carcinoma

**DOI:** 10.1016/j.jdcr.2023.07.012

**Published:** 2023-07-20

**Authors:** Yuka Saeki, Hideaki Miyachi, Keiko Miura, Daijiro Okazaki, Makoto Yamamoto, Yumika Yuki, Michiyo Nakano

**Affiliations:** a Department of Dermatology, Asahi General Hospital, Asahi, Chiba, Japan; b Department of Dermatology, Graduate School of Medicine, Chiba University, Chiba, Chiba, Japan; c Department of Surgical Pathology, Tokyo Medical and Dental University Hospital, Bunkyo-ku, Tokyo, Japan

**Keywords:** mycosis fungoides, pseudocarcinomatous hyperplasia, squamous cell carcinoma, verrucous psoriasis

## Introduction

Mycosis fungoides (MF), the most common type of cutaneous T-cell lymphoma (CTCL), presents with various clinical and histopathological manifestations. When associated with pseudocarcinomatous hyperplasia (PCH), marked keratinization and verrucous lesions that mimic verrucous carcinoma (VC) can appear. Reports of PCH associated with MF are scarce and misdiagnosis as VC or squamous cell carcinoma (SCC) can occur.^1^ We present a case of MF with PCH that required a reevaluation of the initial diagnosis.

## Case report

An 86-year-old man presented with diffuse erythema accompanied by multiple verrucous nodules over his entire body. The patient had persistent erythema for approximately 50 years; however, he had not sought medical attention in the past 40 years, and had no significant medical history nor received phototherapy. One year before his first visit to our clinic, multiple verrucous nodules with keratinization had appeared that were gradually enlarging, particularly on the bilateral wrists, ankle joints, and thighs. Nine months later, the patient noticed a giant mass in the right lumbar region.

**Fig 1:**
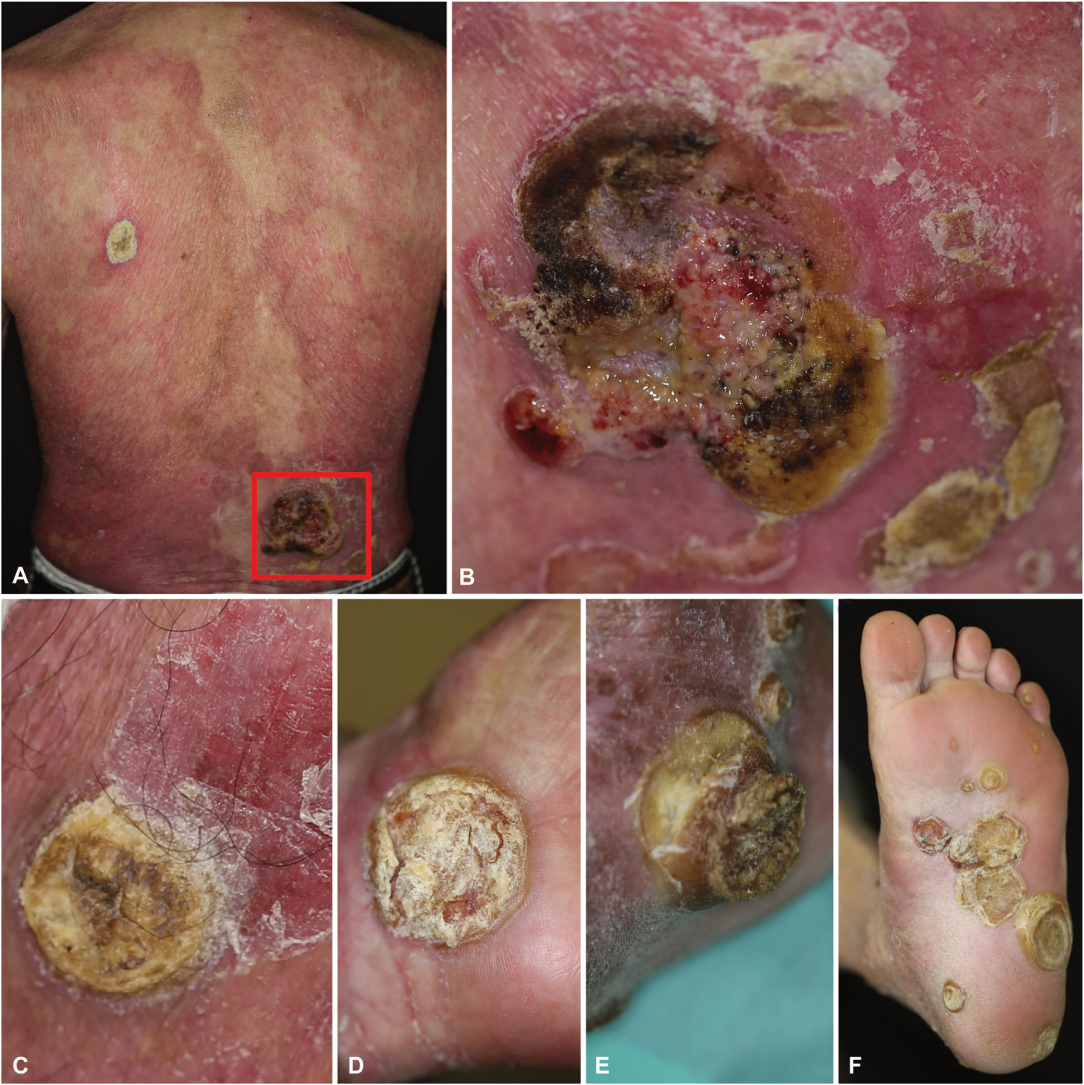
Diffuse erythema of the back (**A**) and a tumor (*red square*) with ulceration and necrosis in the right lumbar region (**B**). Multiple verrucous nodules on the limbs with marked keratinization (**C-F**).

On examination, erythroderma with marked desquamation and multiple keratotic verrucous nodules on the extremities and back were observed (Fig 1, *A-F*). The most significant lesion was in the right lumbar region; a 10 cm wide erythematous tumor with central ulceration and necrosis (Fig 1, *B*). A high soluble interleukin 2 receptor level of 2030 U/mL (normal limit, 157-474 U/mL) was also noted. Positron emission tomography-computed tomography showed hypermetabolism in multiple cutaneous nodules throughout the body, with a maximum standardized uptake value of 14.2 in the right lumbar mass. Additionally, mild lymph node enlargement was detected in the mediastinum, axilla, inguinal, and hilar regions, but the size and standardized uptake value were suggestive of a reactive nature.

Biopsies of the verrucous nodules on the thighs showed papillomatous proliferation with marked epidermal keratinization, parakeratosis, partial loss of the granular layer, and elongation of the rete ridges. Biopsy of the right lumbar mass revealed proliferation of large atypical cells with rounded and unequally enlarged nuclei and basophilic cytoplasm in the epidermis. Mitotic figures were also observed. The patient was diagnosed with verrucous psoriasis (VP) and VC of the right lumbar region. A total excision of the lumbar mass was subsequently performed.

**Fig 2:**
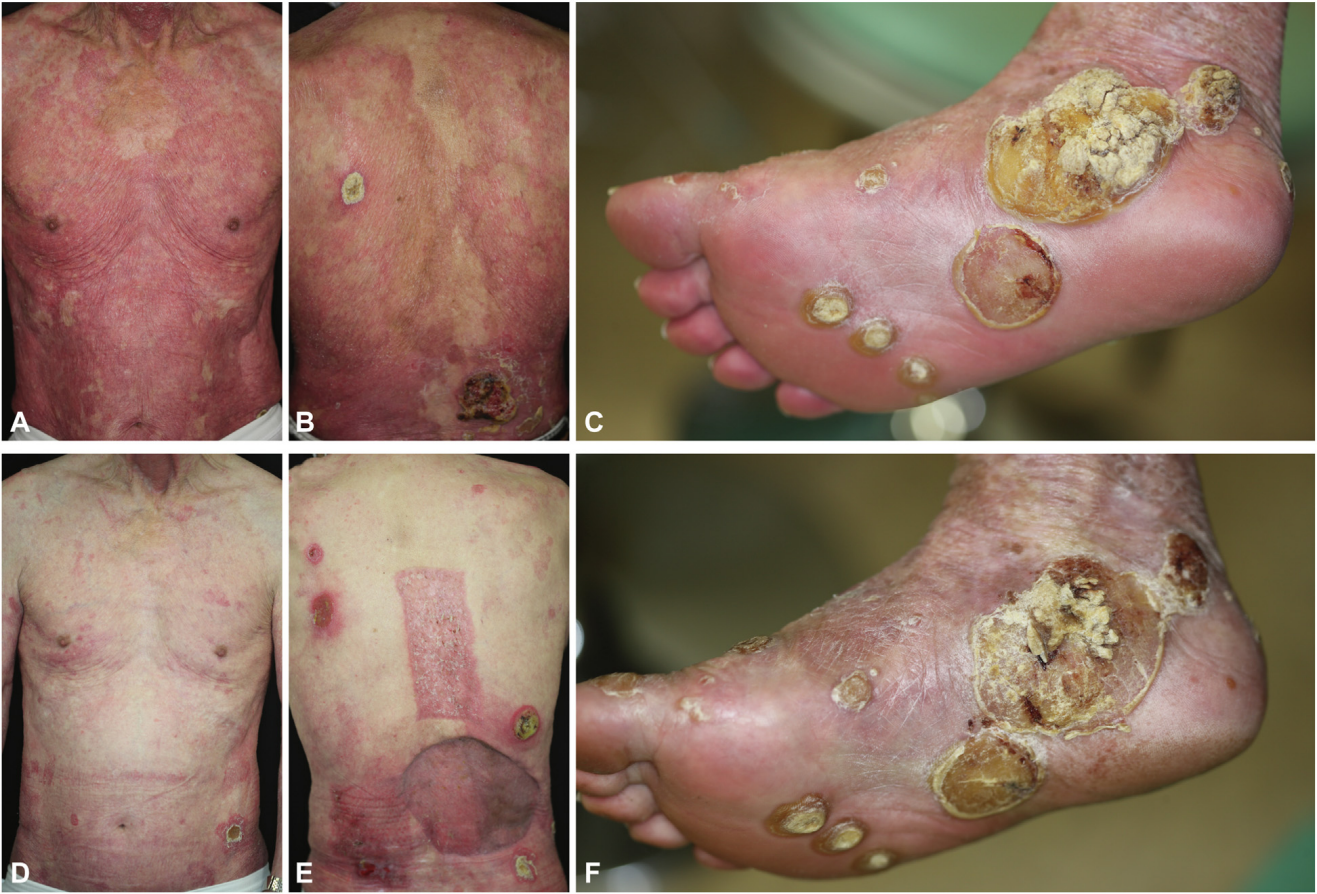
Erythroderma with keratotic verrucous nodules at baseline (**A-C**). One month after starting treatment with etretinate, remission of the erythroderma was observed; however, the treatment was ineffective for the keratotic verrucous nodules (**D-F**).

High-potency topical corticosteroids and etretinate (maximum dose of 30 mg/d) were selected to treat the remaining VP-diagnosed skin lesions (Fig 2, *A*-*C*). After 1 month, the erythroderma had improved, supporting the VP diagnosis; however, after 4 months the treatment was deemed ineffective for the keratotic verrucous nodules, calling the initial diagnosis into question (Fig 2, *D*-*F*). Before considering further treatments with immunosuppressive agents or biologics for VP, the histopathological specimens were reevaluated for dermatopathology.

**Fig 3:**
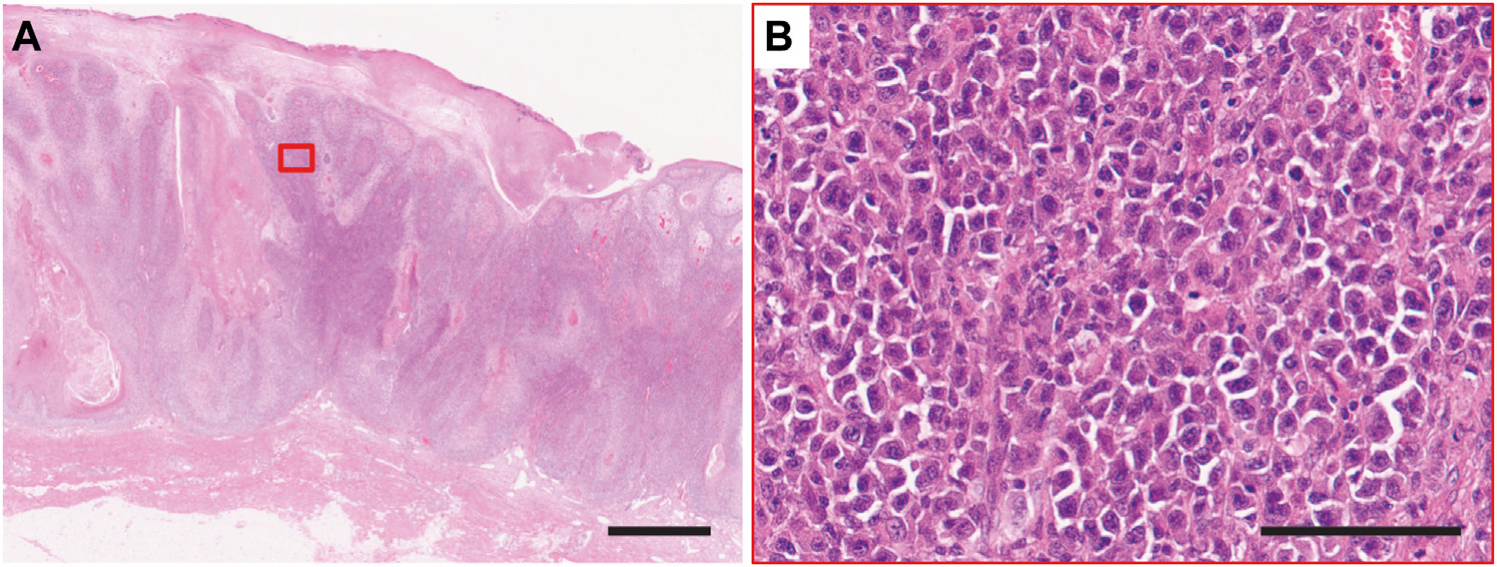
Low-power view of a hematoxylin and eosin-stained section showing that the tumor cells formed nests in the dermis (**A**, scale bar: 200 μm). High-power view of a hematoxylin and eosin-stained section demonstrating that the tumor cells had no apparent intercellular bridges, the nuclei were unequal in size, and mitotic figures were evident (**B**, scale bar: 100 μm). The *red square* indicates the area observed in the high-power view.

**Fig 4:**
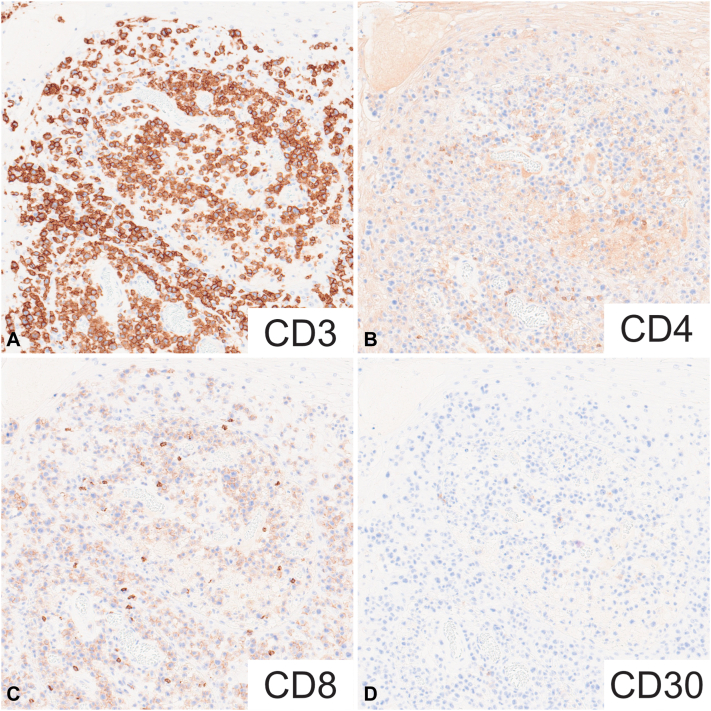
Immunohistochemical staining reveals atypical infiltrating mononuclear cells with expression of CD3 (**A**). CD3-positive T-cells showed the loss of CD4 (**B**), CD8 (**C**), and CD30 (**D**) expression.

On reevaluation, the lack of intercellular bridges between atypical cells and negative cytokeratin AE1/AE3 staining of these cells indicated a misdiagnosis (Fig 3). The atypical cells were determined to be of T-cell origin and positive for CD3 and CD7 but negative for CD4, CD8, CD30, CD56, and T-cell receptor (TCR)-δ (Fig 4). Cytotoxicity markers, granzyme B and T-cell intracellular antigen-1, were positive, and the patient tested negative for the human T-cell lymphotropic virus type 1 virus. TCR rearrangements were positive for TCR-γ and TCR-β, supporting a diagnosis of lymphoma. Cytokeratin AE1/AE3-positive keratinocytes surrounding the atypical lymphoid cells mimicked SCC; however, the lack of penetrating elastic fibers, as examined by Elastica van Gieson staining, was compatible with PCH.^2^ Therefore, we diagnosed the patient with CD4/CD8 double-negative MF accompanied by PCH lesions. No lymph node involvement or visceral lesions were detected. Owing to his refusal for chemotherapy and his advanced age, the patient was placed on follow-up without specific treatment for MF.

## Discussion

MF presents as erythema, plaques, and tumors, with varying clinical and histopathological characteristics. In the early stages, MF often resembles inflammatory skin diseases such as atopic dermatitis, psoriasis, and chronic eczema.^3^ Representative CTCL with keratinization and ulceration includes tumor stage MF, primary cutaneous aggressive epidermotropic cytotoxic CTCL, primary cutaneous anaplastic large cell lymphoma, and primary cutaneous γδ CTCL.^4^^,^^5^ However, MF accompanied by PCH is difficult to diagnose because of its rarity.

The tumor cells of the present case were CD4/CD8 double-negative, which is atypical for MF, although such characteristics have been reported in both early and advanced stages of the disease.^3^ Granzyme B and T-cell intracellular antigen-1 expression reportedly increases with MF progression to the tumor stage, consistent with our case.^6^ Furthermore, T-cell intracellular antigen-1 expression has been reported in cases of CD4/CD8 double-negative MF.^7^

Distinguishing PCH from well-differentiated SCC can be challenging, especially when only a superficial biopsy or examination of a histopathological specimen under scanning magnification are performed.^8^ PCH is a reactive proliferation of the epidermis that is associated with many disorders. Keratinocytes of PCH may be atypical and have mitotic figures but these histopathological findings are less prominent than those of SCC.^1^

A study summarizing 120 cases of PCH associated with lymphoproliferative disorders from the literature showed that most of the cases were associated with CD30-positive lymphoproliferative diseases, while only 3.3% were diagnosed with MF.^1^ Furthermore, 15 of the 120 cases were initially histopathologically diagnosed as SCC. Our patient was also misdiagnosed with VP and VC, the latter being a variant of SCC. The clinical presentation and original histopathological examination of hematoxylin and eosin-stained specimens were consistent with the initial misdiagnosis of VP and VC. Patients with psoriasis have a higher risk of developing malignancy including SCC, especially after receiving phototherapy, than individuals without psoriasis.^9^ Reevaluation of the diagnosis was further delayed owing to a partial response to the etretinate chosen to treat the VP lesions because retinoids are partially effective for CTCL.^10^ Thus, dermatologists should be cautious in the diagnosis and follow-up of similar cases, even when the initial treatment appears to be effective.

In conclusion, we report a case of PCH associated with MF that was initially misdiagnosed as SCC. This case serves to remind dermatologists of the diverse clinical and histopathological presentations of MF. Furthermore, it emphasizes the importance of careful evaluation for the accurate diagnosis of PCH or SCC in patients with MF to reduce the risk of misdiagnosis and delay in treatment initiation.

## Conflicts of interest

None disclosed.
